# The COPHELA (Cooperation in Quality Assurance for Pharmacy Education and Training between Europe and Latin America) Project

**DOI:** 10.3390/pharmacy8010029

**Published:** 2020-03-04

**Authors:** Antonio Sánchez-Pozo, Afonso Miguel Cavaco, Paolo Blasi, Mariana Ortiz Reynoso, Carlos Tomas Quirino-Barreda, Patricia Acuña Johnson, Fernando Torres Moscoso, Selma Rodrigues de Castilho, Claudia Fegadolli, Sergio Slan Zarwar, Jeffrey Atkinson, on behalf of the COPHELA Associated Partners

**Affiliations:** 1 Facultad de Farmacia, Campus Cartuja, Universidad de Granada-UGR, 18701 Granada, Spain; sanchezp@go.ugr.es; 2 Social Pharmacy Department, Faculdade de Farmacia, Universidade de Lisboa – FFUL-Lisboa, 1649-003 Lisbon, Portugal; acavaco@ff.ulisboa.pt; 3 Dipartimento di Farmacia e Biotecnologie, Alma Mater Studiorum – Università di Bologna, 40127 Bologna, Italy; paolo.blasi@unicam.it; 4 Facultad de Farmacia, Universidad Autónoma del Estado de México – UAEM, Toluca 50000, Mexico; marianaor@yahoo.com; 5 Facultad de Farmacia, Universidad Autónoma Metropolitana Xochimilco - UAM-X, Mexico City 14387, Mexico; cquirino@correo.xoc.uam.mx; 6 Facultad de Farmacia, Universidad de Valparaíso – UV, Valparaíso 2362735, Chile; patricia.acuna@uv.cl; 7 Escuela de Química y Farmacia, Facultad de Medicina, Universidad Andrés Bello – UNAB, Santiago de Chile 8370146, Chile; ftorres@unab.cl; 8 Faculdade de Farmacia, Universidade Federal Fluminense – UFF, Niterói 24241000, Brazil; selmarc@id.uff.br; 9 Faculdade de Farmacia, Universidade Federal de São Paulo – UNIFESP, São Paulo 04021-001, Brazil; cfegadolli@unifesp.br; 10 Departamento de Gestão do Ambiente Educacional, Racine Qualificação e Assessoria – Rua Padre Chico 93, Pompéia IR, São Paulo CEP 05008-010, Brazil; sslan@racine.com.br; 11 Université de Lorraine-UL, 12 rue de Versigny, 54600 Villers, France

**Keywords:** pharmacy, education, specialization, master degree, distance learning, European Union, Latin America, cooperation, patient safety, pharmaceutical industry

## Abstract

COPHELA (Cooperation in Quality Assurance for Pharmacy Education and Training between Europe and Latin America), a collaborative project between the European Union (EU) and Latin America, will produce on-line courses for the master degree in pharmacy. The program runs from 2019 through 2021. It is funded by the Erasmus+ program of the Education, Audio-visual and Culture Executive Agency (EACEA) of the European Commission. The partners are EU and Latin American universities. These are accompanied by associated partners from EU and Latin American universities, as well as from governmental and non-governmental organizations, such as pharmacy chambers and educational associations. The project is coordinated by the University of Granada, Spain (first author of this paper). It will produce distance learning master degree courses in a dozen fields of specialized pharmaceutical science education and practice, ranging from patient care to industrial pharmacy. This paper describes the design of the project and is intended to evoke constructive comments. It also represents a call for the recruitment of additional associated partners.

## 1. Introduction

In light of the evolving situation in pharmacy specialization in Latin America, the COPHELA project proposes to produce distant learning tools for master degrees in pharmacy specialization. These will be published in Spanish, Portuguese and/or English, and will be available primarily for the Latin American market but also in the EU.

This COPHELA project is a follow-on to three previous EU-funded projects:

1. PHARMINE (Pharmacy Education in Europe) [1,2], and the derivatives of PHARMINE viz
2. PHAR-QA (Quality Assurance in European Pharmacy Education and Training) [3,4], and
3. PHAR-IN (Competences for industrial pharmacy practice in biotechnology) [5,6].

All three projects centered on:

1. The competences for fundamental and specialized pharmacy practice (community, hospital and industry)
2. The education and training tools necessary to produce the competences cited previously in (1) above, and
3. The harmonization of competences, and the education and training tools necessary, in the EU member states.

The purpose of the three previous projects was to adapt pharmacy education in the EU to the on-going evolution in practice. For example, community and hospital pharmacies are moving towards patient care activities, such as therapeutic monitoring, intervention in public health campaigns (vaccination and other), medicinal treatment of long-term ailments, and interpretation of clinical biological results, etc. There is also a substantial transformation in industrial pharmacy in research and development, production, etc. This accompanies the shift in therapeutics from small chemical drug molecules to large protein biosimilars. The development of the generic drug market with the consequential changes in marketing, regulatory affairs, etc., is another example of the evolution in practice. 

COPHELA looks at the paradigms and technology required to extend to Latin America the principles behind the three EU PHARMINE projects. It aims at creating master degree courses in specialized pharmaceutical sciences to meet the challenges of the changing face of pharmacy. Below, we examine the characteristics of pharmacy practice in the EU and Latin America, and how specialized education and training can provide possibilities for adaptation in the changing world.

### 1.1. Characteristics of Pharmacy Practice

The attributes of pharmacy practice, with examples from the EU and Latin America, are:

1. **Workplace.** A hospital pharmacist can be defined as a pharmacist working in a hospital pharmacy -and this independently of the education and training s/he has followed. An industrial pharmacist can be defined as a pharmacist working for a drug company. In both cases –hospital and industrial – this attribute is independent of the following three attributes.
2. **Specific pharmaceutical activities.** This concerns the definitions of the practice of community and hospital pharmacy (and to a lesser extent industrial pharmacy). Such definitions involve pharmaceutical acts like formulation of specific forms of drugs or deliverance of specific drug treatments. The practitioners’ perceptions of such acts have been described in detail [7,8,9].
3. **Laws and directives.** The definition of attribute 2 –pharmacy practice –is established through laws and directives from national associations and/or chambers, and, in the case of the EU, by directives drafted by the European Commission and approved by the European Parliament. It should be noted that a directive is an act that requires a given country to achieve a result without dictating the means of achieving that result. Thus hospital pharmacy in Spain and Portugal are governed by associations and chambers [10,11], and identified as specific activities by national law [12,13]. At the pan-European level, an EU directive [14] defines pharmacy practice (and education), setting out rulings on subject matters such as patient care, this EU directive does not mention hospital pharmacies as such. With respect to industrial pharmacy practice and education, the EU directive again does not acknowledge the existence of industrial pharmacy specialization, but does specify that subjects, such as “manufacture and testing of medicinal products” are part of the “professional experience” of the pharmacist. At a national level, in Spain and Portugal, industrial pharmacy is taught as a master’s degree [15,16,17,18], and industrial pharmacists are represented by specific chambers or associations [19,20]. The study of legislation on pharmacy specialization in Latin America is the first objective of COPHELA.
4. **Education and training.** This attribute concerns the education and training for fundamental practice and for specialized practice in hospital and industrial pharmacy. As an example, in Spain and Portugal, hospital pharmacy practice is taught in specific hospital pharmacy specialization courses [21,22]. Concerning Latin America, the second objective of COPHELA is to create and install education and training for specialized pharmacy practice. It is interesting to note that the recognition of specialization in pharmacy involves the creation of master degree courses, thus evolution in education goes hand in hand with evolution in pharmacy practice [23].

### 1.2. The COPHELA Project

Below, are the paradigms and methodology to achieve these aims. 

## 2. Methodology

The first stage in the elaboration of the COPHELA project was to constitute a core team of 10 partners (authors of this paper) who replied to the following criteria:

- Evidence of collaboration between the EU and Latin America in the form of student and/or staff exchange, joint projects in education and training and/or research, etc.
- Present and future development of specialization in pharmacy practice in the fields of patient care, research in pharmaceutical science and/or education, etc.
- Development of IT capacity in teaching.
- Interaction with national and international governmental departments (education, health …), pharmacy chambers and associations, etc.

**Figure 1:**
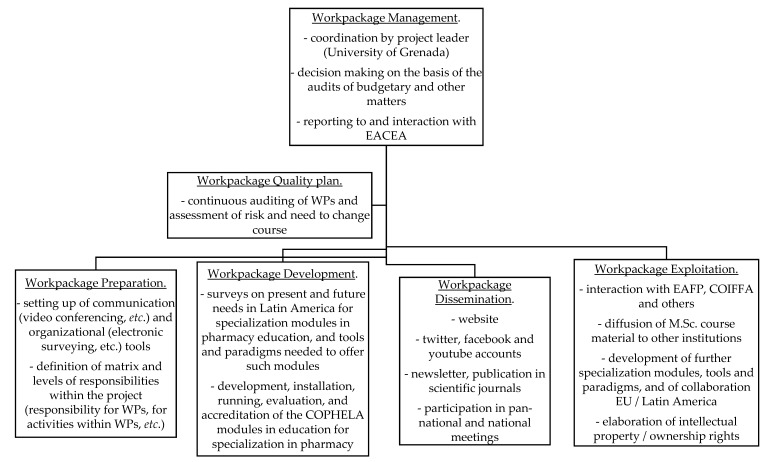
Structure of the COPHELA project. EAFP: European Association of Faculties of Pharmacy [24]; COIFFA: Conferencia Iberoamericana de Facultades de Farmacia [25].

EACEA/EU projects are separated into “workpackages” (WPs) that group the activities of the project. WPs function independently but rely on the results of other WPs. Figure 1 shows the organization of the WPs. WP “management” coordinates the project, whereas WP “quality plan” audits the project. These two WPs operate throughout the project. WP “preparation” sets the stage for the project; WP “development” produces the on-line courses. WP “dissemination” ensures that the courses developed are available to all those concerned, and WP “exploitation” guarantees that the on-line courses are used by all potential partners during, and after, the lifetime of the project. The chronological order of the WPs “preparation” through “exploitation” is shown by the arrow at the bottom of the figure.

## 3. Discussion

The target group and the ultimate beneficiaries of the COPHELA project are the populations of the Latin American countries. Benefit will come in various forms. Firstly, there will be an improvement in the healthcare of the Latin American population, following the amelioration of pharmaceutical care, based on the upgrade of pharmaceutical specialization in community and hospital pharmacies. Secondly, the Latin American pharmaceutical industry will benefit by providing employees with the tools required for future changes in the industry, such as the change from a chemistry-based industry producing small molecules to a biotechnology-based industry producing large therapeutic proteins.

There are several potential issues. Firstly, they involve shortcomings in the performance of the partners and their capacity to produce, evaluate and accredit satisfactory courses. in the time given. Appropriate expedient measures to counter such deficiencies are based on the capacity of the auditing to detect and suggest solutions, and a satisfactory interaction with EACEA and project partners.

Secondly, problems could arise in the accreditation of courses by bodies external to the project such as government health and education authorities, and thus, in the long-term use of COPHELA material post-2021, the official date of the closure of the project. The main purpose of COPHELA accreditation is recognition. The project in its present form has 44 partners and associated partners in the EU and Latin America. The fact that all the COPHELA universities and other institutions have contacts with the accreditation authorities in their different countries should ensure that such problems can be resolved. 

Thirdly, in parallel to accreditation, problems could arise concerning intellectual property and ownership of the material produced given that in the EU and in Latin America the economics of the education and training of pharmacists vary greatly. This ranges from the situation in state-owned universities where such education and training are without charge, to that in private universities with a fee system. Here, solutions will be found on the basis of interactions between the project partners and associated partners, and the relevant partners in the healthcare system of the country concerned. Ownership will be guaranteed by the commitments and conventions on a joint project. Intellectual property rights will be ensured through Creative Commons Licenses [26].

## 4. Conclusions

COPHELA is a collaborative project between the EU and Latin America aimed at improving specialization education in pharmacy. Its ambition is to improve not only the competences of target groups, such as university staff and students, and pharmaceutical professionals, but also the wellbeing and prosperity of the general public through better patient care, and better drugs, with a modernized pharmaceutical industry capable of providing the latter. The purpose of this manuscript is to request comments on project design and recruit potential research partners.

## Appendix A

**Associated partners/Universities:**

**Spain:**

Universidad de Alcalá, Faculty of Pharmacy – UAH, Alcala de Henares, Madrid, Spain. http://www.uah.es/;

Universidad de Salamanca, Faculty of Pharmacy – USAL, Salamanca, Spain. http://www.usal.es/webusal/en/node/24;

Universidad Complutense de Madrid, Facultad de Farmacia – UCM, Madrid, Spain. http://www.ucm.es/;

Universidad de Santiago de Compostela, Facultad de Farmacia – USC, Santiago de Compostela, Spain. http://www.usc.es/;

Universidad de Valencia, Facultad de Farmacia – UV, Valencia, Spain. http://www.uv.es/;

**Portugal:**

Universidade de Coimbra Facultade de Farmacia, Coimbra, Portugal – UC. http://www.uc.pt/;

**Italy:**

Università degli Studi di Perugia – UNIPG, Perugia, Italy. http://www.unipg.it/en/;

Università degli Studi Guglielmo Marconi – UNIMARCONI, Rome, Italy. www.unimarconi.it;

**Brazil:**

Universidade de São Paulo, Faculty of Pharmaceutical Sciences – USP, Sao Paulo, Brazil. http://www5.usp.br/;

**Argentina:**

Universidad Nacional de Córdoba, Department of Pharmacy of the Chemical Science Faculty – UNC, Cordoba, Argentina. http://www.unc.edu.ar/;

Universidad Nacional de San Luis, Facultad de Quimica, Bioquimica y Farmacia – UNSL, San Luis Argentina. http://www.unsl.edu.ar/;

**Peru:**

Universidad Antonio Guillermo Urrelo – UPAGU, Cajamarca, Peru. http://upagu.edu.pe/es/;

Universidad Norbert Wiener – UWIENER, Lima, Peru. http://www.uwiener.edu.pe/;

**Costa Rica:**

Universidad de Costa Rica – UCR, San José, Costa Rica. http://www.ucr.ac.cr/;

**El Salvador:**

Universidad José Matías Delgado – UJMD, San Salvador, El Salvador. http://www.ujmd.edu.sv/;

**Ecuador:**

Universidad Nacional de Chimborazo/Escuela Superior Politecnica del Chimborazo – ESPOCH, Riobamba, Ecuador. http://www.espoch.edu.ec/;

**Paraguay:**

Universidad Nacional de Asunción – UNA, Asuncion, Paraguay. https://www.una.py/;

**Venezuela:**

Universidad de Los Andes – ULA, Merida, Venezuela. http://www.ula.ve/;

Associations and chambers.

**Europe:**

Real Academia Nacional de Farmacia – RANF, Madrid, Spain. http://www.ranf.com/;

Consejo General de Colegios Oficiales de Farmacéuticos de España – REDFARMA, Madrid, Spain. http://www.portalfarma.com/profesionales/organizacionfcolegial/Paginas/organizacionfarmaceuticacolegial.aspx;

Academia de Farmacia de Castilla y León, Salamanca, Spain. http://www.academiadefarmaciacastillayleon.es/;

Academia Iberoamericana de Farmacia – NSACAN, Grenada/Seville, Spain. http://www.insacan.org/aif/aif.html;

Asociación Universitaria Iberoamericana de Postgrado – AUIP, Salamanca, Spain. http://www.auip.org/;

Sociedad Española de Farmacia Hospitalaria – SEFIG, Madrid, Spain. http://www.sefh.es/;

Istituto Italo-Latino Americano, Rome, Italy. http://www.esteri.it/mae/en/politica_estera/aree_geografiche/americhe/istitutoitalolatinoamericano;

European Association of Hospital Pharmacy – EAHP, Brussels, Belgium. http://www.eahp.eu/;

European Association of Faculties of Pharmacy – EAFP, Msida, Malta. http://eafponline.eu/;

European Pharmacy Students Association – EPSA, Brussels, Belgium. http://www.epsa-online.org/;

**Latin America:**

Academia de Ciências Farmacêuticas do Brasil – ACADEMIAFARMACIA, Rio de Janiero, Brazil. http://www.academiafarmacia.org.br/;

Asociación de Quimicos Farmaceuticos del Paraguay, Asuncion, Paraguay.

Conferencia Iberoamericana de Facultades de Farmacia – COIFFA, Mexico City, Mexico. http://www.coiffa.org.mx/;

Unión de Universidades de América Latina y el Caribe – UDUAL, Mexico D.F., Mexico. www.udual.org;

Erasmus Mundus Association Latin America Chapter – EMA, Mexico D.F., Mexico. http://www.em-a.eu/en/about-ema/regional-chapters/latin-american-chapter.html.
